# Hemiarthroplasties in young patients with osteonecrosis or a tumour of the proximal femur; an observational cohort study

**DOI:** 10.1186/1471-2474-14-31

**Published:** 2013-01-17

**Authors:** Pim W van Egmond, Antonie HM Taminiau, Huub JL van der Heide

**Affiliations:** 1 Department of orthopaedic surgery, Leiden University Medical Centre, Albinusdreef 2, P.O.box 9600, 2333, ZA, Leiden, The Netherlands

**Keywords:** Bipolar, Unipolar, Hemiarthroplasty, Tumour, Osteonecrosis, Young patients, Total hip arthroplasty

## Abstract

**Background:**

The failure scenario in total hip arthroplasty (THA), in younger patients, is dependent on the fixation and wear of the acetabular component. In selected cases, where endoprosthetic replacement of the femoral head is unavoidable for limb salvage or functional recovery, hemiarthroplasty can be chosen as an alternative. The purpose of this study is to evaluate hemiarthroplasty as treatment strategy for young patients with osteonecrosis or a tumour of the proximal femur.

**Methods:**

Between 1985 and 2008, 42 hemiarthroplasties (unipolar and bipolar) were performed in patients younger than 65 years with osteonecrosis (n=13) or a tumour of the proximal femur (n=29). All patients were seen at yearly follow-up examination and evaluated. Revision or conversion to a THA was regarded as a failure of the implant. A Kaplan Meier analysis was performed. To determine significant differences between categorical groups, the Pearson chi-square test was used. In numerical groups the independent T-test and One-way ANOVA were used.

**Results:**

After a mean follow-up of 7.1 years, failure of the hemiarthroplasty occurred 6 times. The Kaplan Meier survival analysis with conversion to THA or revision as endpoint of the bipolar hemiarthroplasties (n=38) shows a 96% survival at 15, and 60% at 20 years. In the unipolar type (n=4) we found a conversion rate of 50% within 3 years.

**Conclusions:**

Bipolar hemiarthroplasty is a reasonable alternative in a young patient with osteonecrosis or a tumour of the proximal femur as indication. Because of the high conversion rate after unipolar hemiarthroplasties, we would not recommend this type of prosthesis in the young patient.

## Background

Total hip arthroplasty (THA) is one of the most important advances in lower extremity reconstruction of the past century. However, this procedure in young patients is known to fail more frequently than in older patients [1-5]. One of the hypotheses is that the high activity level of these patients increases the risk of wear, debris reaction and mechanical failure of the implant [6]. This is seen especially in acetabular component loosening [7-11]. Because revision of this implant becomes more likely with higher functional demand, hemiarthroplasty might be of benefit in this group. Unipolar or bipolar hemiarthroplasty is almost exclusively used for proximal femoral fractures in the elderly patient [12-20]. Acetabular protrusion is judged to be an important factor causing early failure, or problems at revision [6,21-24]. However literature of this procedure in young patients is scarce and better bone stock might prevent protrusion or acetabular erosion.

End-stage osteonecrosis or oncologic destruction of the proximal femur are similar in that both can be treated by an endoprosthesis and are considered by us as two valid indications to consider hemiarthroplasty in the young patient. Although RCT’s show superior results of THA compared to hemiarthroplasties in the short term and have the tendency to be superior after 7–10 years of follow up; these studies regard fractures in elderly patients with a mean age of >70 years [13-15,25,26].

In this study we reviewed all consecutive hemiarthroplasties from our department in young patients, both unipolar and bipolar, with osteonecrosis or tumour resection as indication.

## Methods

This study comprises an observational cohort study in patients who received a hemiarthroplasty, bipolar (n=38) or unipolar (n=4) with osteonecrosis of the femoral head (n=13), or a proximal femur resection for a tumour (n=29). The medical records and radiographs of the patients included at the Leiden University Medical Centre between 1985 and 2008 were reviewed. Radiographic evaluation for acetabular erosion was measured following the classification proposed by Baker et al [14].

Revision or conversion to a THA was regarded as a failure of the implant. A Kaplan Meier survival analysis was performed for both unipolar and bipolar arthroplasties with conversion to THA or revision as endpoint. A COX regression analysis was used to determine the independent effects of variables. To determine significant difference between categorical groups, the Pearson chi-square test was used. In numerical groups the independent T-test and One-way ANOVA were used. An alpha value of 0.05 was used as level of significance. All statistical analyses were performed using SPSS version 17.0 for Windows (SPSS Inc., Chicago, IL).

Approval of an ethics committee is not necessary in observational research in the Netherlands.

## Results

In the period between 1985 and 2008, 42 hemiarthroplasties were performed in 39 patients with osteonecrosis grade IV or a tumour in the proximal femur. Sex, side and diagnosis are shown in Table 1. The average age at the time of operation was 39 years (SD=15, Range 13–66). All patients were operated at the Leiden University Medical Centre, by orthopaedic surgeons with experience in endoprosthetic reconstruction of the hip.

**Table 1:** Implants descriptive; subdivided into unipolar and bipolar hemiarthroplasties

			**Hemiarthroplasty**	
		**N(implants)**	**Unipolar**	**Bipolar**
**Indication**	Tumour	29	1	28
	Osteonecrosis	13	3	10
**Sex**	Man	23	3	20
	Woman	19	1	18
**Side**	Left	22	0	22
	Right	20	4	16
**Total**		**42**	**4**	**38**

The mean follow up was 7.1 years (SD=6, Range 1–25). Fifteen patients (15 implants) died, 14 from the sequelae of their primary tumour, during follow up, 1 in the unipolar group and 14 in the bipolar group. From the deceased patients, none had a revision or conversion before the time of death. This did not differ significantly between the unipolar and the bipolar group (p=0.427).

In our series osteonecrosis caused by high dose corticosteroid use was seen in 5 patients (8 implants), in one case by alcohol abuse, in two cases by an earlier operation or trauma of that limb and in two cases a cause could not be found. Corticosteroid therapy was in each case started as part of treatment in leukaemia. All patients had a grade IV osteonecrosis according to Steinberg’s classification [27]. Tumours were malignant in 24 cases and benign in 2 cases; in the remaining three cases a metastasis of breast cancer in the proximal femur was the indication for resection. Ten cases, including the three metastases of breast cancer, had a metastasis in follow up or at presentation (Table 2).

**Table 2:** Type of tumour and presence of metastasis at time of treatment or during follow-up

	**Tumour**		**Metastasis**	
		**N(cases)**	**Yes**	**No**
**Malignant**	Osteosarcoma	13	4	9
	Chondrosarcoma	8	2	6
	Ewing sarcoma	3	1	2
**Benign**	Giant cell tumour	1	-	1
	Chondroblastoma	1	-	1
**Metastasis**	Breast cancer	3	3	-
**Total**		**29**	**10**	**19**

The different types of prostheses used, each in its respectable time-span, were the considered first choice at that time at our department (Table 3). As a significant difference in prosthetic survival occurred between the unipolar and bipolar hemiarthroplasties, we will consider them as separate groups. From the 42 femoral stems, one (a bipolar implant) was revised because of a pseudo-arthrosis of the allograft-femoral junction, and was converted to a THA after 4 years. No acetabular erosion was noticed. Two bipolar implants were converted to a total hip arthroplasty after 7.5 and 23.7 years and one bipolar head was exchanged after a mechanical failure of the locking ring of the bipolar head after 15.1 years. In the two patients with bipolar hemiarthroplasties converted to a THA, acetabular erosion was objectified during operation (Table 4).

**Table 3:** Type of prostheses and technique (cemented/uncemented)

**Hemiarthroplasty**			**Cemented**	
		**N(implants)**	**Yes**	**No**
**Unipolar**	Mallory Head	3	-	3
	Cemented NOS*	1	1	-
**Bipolar**	Mallory Head	28	5	23
	Mutars	5	2	3
	Lord/Kotz	4	-	4
	Cemented NOS*	1	1	-
**Total**		**42**	**9**	**33**

*NOS; Not Otherwise Specified.

**Table 4:** Failed implants

**Failed implants**	**Hemiarthroplasty**	**Type / Technique**	**Indication**	**Age at time of operation**	**Time till failure (years)**	**Complaints / diagnosis failure of implant**	**Procedure**
A	Unipolar	Mallory Head/Uncemented	Osteonecrosis	23	1.0	Pain in groin region, positive reaction on bupivacain	Conversion to THA
B	Unipolar	Mallory Head/Uncemented	Osteonecrosis	54	1.3	Pain in groin region, positive reaction on bupivacain	Conversion to THA
C	Bipolar	Mallory Head/Uncemented	Tumour	33	4.2	Radiographic loosening, per operative pseudo-arthrosis of the allograft-femoral junction	Conversion to THA
D	Bipolar	Mallory Head/Uncemented	Tumour	21	7.5	Pain in groin region, per operative acetabular erosion	Conversion to THA
E	Bipolar	Mallory Head/Uncemented	Tumour	48	15.1	Unexplained feelings/pain; per operative: mechanical failure of part of implant	Revision head of bipolar implant
F	Bipolar	Lord/Kotz/Uncemented	Tumour	35	23.7	Pain in groin region, per operative acetabular erosion	Conversion to THA

In the unipolar group 2 conversions to a THA were performed because of pain in the groin region, after a positive reaction on bupivacain. Both patients had evidence of acetabular erosion during operation (Table 4, Table 5).

**Table 5:** Implant survival and grade of acetabular erosion, subdivided into unipolar and bipolar hemiarthroplasties

			**Hemiarthroplasty**	
		**N(implants)**	**Unipolar**	**Bipolar**
**Implant**	In situ	36	2	34
	Conversion/Revision	5/1	2/0	3/1
**Acetabular erosion**	Grade 0	27	1	26
	Grade 1	13	3	10
	Grade 2	2	0	2
	Grade 3	0	0	0
**Total**		**42**	**4**	**38**

**Figure 1:**
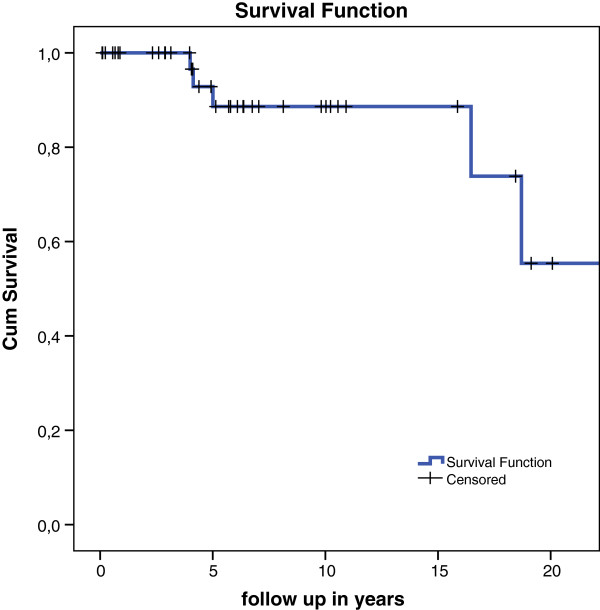
Kaplan Meier survival plot for uni- and bipolar hemiarthroplasties with conversion or revision as endpoint.

**Figure 2:**
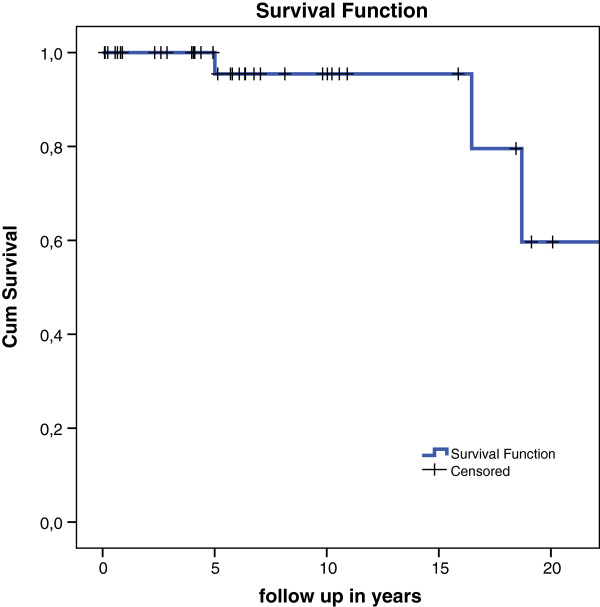
**Kaplan Meier survival plot of only bipolar hemiarthroplasties with conversion or revision as endpoint.** A survival plot of only unipolar hemiarthroplasties was not deemed valuable because of the small number of unipolar implants.

Apart from the higher failure rate in unipolar implants, they also failed faster, with a mean survival of only 1 year. Table 5 lists the specific cases of implant failure. Due to the small amount of unipolar prostheses in this series a Kaplan Meier plot of this group alone is not useful. When we combine the groups uni- and bipolar prostheses a Kaplan Meier plot shows a survival rate of 89% after 15 and 56% after 20 years (Figure 1). The Kaplan Meier analysis for the bipolar prostheses alone shows a survival rate of 96% after 15 years. After 20 years this is reduced to 60% (Figure 2). The unipolar prostheses have a survival rate of only 50% after nearly 2 years. A COX regression analysis showed no statistically significant difference in side operated, indication, sex, bipolar or unipolar hemiarthroplasty and technique (cemented or uncemented) used. There was no significant difference in the specific type or brand of prostheses used and failure of the implant.

Other complications requiring a second surgery, but not revision of the implant, were: two infections treated with debridement and antibiotics (systemically and locally), two dislocations including one of the patients with an infection, were treated by a closed reduction and were stable afterwards.

Radiographic evaluation showed acetabular erosion in fifteen implants. Of these, thirteen were grade I (narrowing of articular cartilage, no bone erosion) and only two a grade II (acetabular bone erosion and early migration); protrusio acetabuli (grade III) was not seen (Table 5).

## Discussion

### Total hip arthroplasty versus hemiarthroplasty

Total hip arthroplasty in the elderly is a safe and effective procedure with a survival rate as high as 90% after 15 and 75% after 25 years [1]. Whereas, in older patients, the majority of patients are treated with a THA because of primary osteoarthritis, the indication for an arthroplasty in young patients varies and includes secondary osteoarthritis (most commonly secondary to developmental dysplasia of the hip or trauma), osteonecrosis, ankylosing spondylitis, juvenile idiopathic arthritis, epiphyseal dysplasia, sequelae of Perthes disease, chondrodystrophica and fractures. Although RCT’s show superior results of THA compared to hemiarthroplasties in the short term and have the tendency to be superior after 7–10 years of follow up; these studies regard fractures in elderly patients with a mean age of >70 years [13-15,25,26].

Young patients (under 50 years of age) have a much higher implant failure of THA, especially the acetabular component [1-5]. Several studies showed the revision rate, after a THA due to aseptic loosening of the acetabular component to be between 20 and 63% after 10 to 22 years [7-11]. the revision rate for femoral stem loosening however, was between 0 and 23% after 10 to 22 years. Publications from the same institute showed a better 10 year survival of 88% with impaction bone grafting when an acetabular defect was present in combination with cemented cups [5,28,29].

As the femoral component is less likely to fail in young patients, it can support the hypothesis that endoprosthetic survival in the younger patients is longer with hemiarthroplasties. The reported revision rate of bipolar hemiarthroplasties in young patients is between 7-21% after 6–14 years [24,30,31]. This concurs with our series, in which the femoral component had to be revised in one case because of pseudo-arthrosis at the femoral-allograft junction. Dislocation is a serious complication more often seen after THA compared to hemiarthroplasties, especially in the elderly group after a femoral neck fracture [32]. In our group 3 arthroplasties dislocated.

Our results indicate that a bipolar hip replacement, with 96% survival after 15 and 60% after 20 years independent of age or underlying disease, can be superior to a THA in young patients. In the unipolar group, however, the survival rate was only 50% after 2 years.

One of the major mechanisms of failure after hemiarthroplasty is protrusion of the metal head as the acetabular articular cartilage degenerates [6,21-24]. In rare cases even osteolysis of the acetabulum is seen [33].

The degeneration of the articular cartilage is believed to be influenced by, mostly, activity level [6]. The histological process of this degeneration begins with abnormal stress to the articular cartilage due to the hard bipolar cup. This facilitates the secretion of degenerative enzymes which induces the loss of initial glucosaminoglycan. The articular cartilage softens and loses elasticity. Collagen fibres are destroyed and the surface integrity changes. This process is correlated with activity (repetitive stress) levels and the duration of articulation of the implant with the acetabulum [6]. In the end the head will migrate through acetabular cartilage, which is a major cause of the failure of (bipolar) hemiarthroplasty.

Several studies regarding the improvement in functional status after a conversion to a THA after a failed hemiarthroplasty found excellent results [34-36]. When these conversions to a THA were followed in time, they showed a better survival rate of the acetabular component as compared to the femoral component [34,37]. One author reported a reoperation rate of only 4.5% for aseptic loosening of the acetabular component after10 years [35].

We hypothesize these results are explained by the repetitive stress caused by the hard bipolar head. This stress not only causes degeneration of articular cartilage, but also causes the subchondral bone to harden. This process, well known in osteoarthritis, might make the acetabulum component less vulnerable to loosening when conversion to a THA is necessary [38,39]. Especially in the young and active patients in our series, the acetabulum is almost always without damage. Subsequently the subchondral bone should be softer than in patients who have suffered abnormal stress levels and this, we hypothesize, might be the cause for the high rate of aseptic loosening of the acetabulum in THA’s in young patients. We could not confirm this in the current literature and further study should be conducted to explore this hypothesis. In our series we did not encounter problems of protruding bipolar heads.

Hemiarthroplasties produce abnormal stress levels on the acetabulum which in turn causes degeneration [21,40]. However, when this degeneration leads to pain in the patients or complications, or even failure, of the implant; differs from patient to patient. Extensive follow up, both clinical and radiographical, should be advised.

Two authors studied the clinical outcome after bipolar hemiarthroplasties. In both a correlation was shown between a lower Harris Hip Score and articular degeneration and the incidence of buttock, groin or thigh pain could be used as a marker for failure of the implant. Groin or buttock pain was reported for articular degeneration, whereas thigh pain was believed to be a symptom of loosening of the femoral component or an impending fracture. Both authors suggest early revision or conversion in patients with one of these symptoms [23,36]. The patients in our series with a failed implant reported the same complaints (Table 5).

### Unipolar versus bipolar hemiarthroplasty

Bipolar hemiarthroplasties articulates at two different levels and, due to this dual bearing, is thought to have less acetabular wear. Another advantage of this design would be increased range of motion compared to unipolar implants [18]. A potential disadvantage of the bipolar implants is the risk of polyethylene wear, causing synovitis and loosening of the stem. Several RCT’s have failed to present convincing data on differences in clinical outcome between unipolar or bipolar designs and a Cochrane review in 2010 concluded there is currently not enough evidence to support the use of either unipolar or bipolar prosthesis when performing hemiarthroplasty [12]. Acetabular erosion is thought to be the major factor influencing clinical outcome and reason of revision or conversion. Studies regarding acetabular erosion in patients with hemiarthroplasties show ranges from 2% to 36% for unipolar, and 0% to 26% for bipolar implants [13,14,18,22,41,42]. Baker et al [14]. introduced a grading system for acetabular erosion and reported 66% erosion, mostly grade I, after only 3 years of follow up. A recent study found a much lower percentage in bipolar hemiarthroplasties; with only 14% acetabular erosion (all grade I) after four year of follow up [13]. The same author performed a RCT which concluded equivalent clinical outcome between unipolar or bipolar hemiarthroplasties, but a significantly higher incidence of acetabular erosion in the unipolar group [16]. Again, it should be mentioned that these studies are based on elderly patients. In our study we saw acetabular erosion in 35.7%, mostly grade I, after a mean follow up of 7.1 years, which is longer than the studies mentioned above. Because of the small number of unipolar hemiarthroplasties performed in our study, we could not analyze differences between the two types of hemiarthroplasties.

### Limitations

This study had several limitations. First, the number of patients was small; especially in the unipolar hemiarthroplasty group. Secondly it comprises a heterogeneous group of patients. On the other hand, in both patient groups (osteonecrosis and tumour) the acetabulum was not affected; an important difference with other indications for hip arthroplasty. Also, the tumour population can create a bias because of lower life expectancy after surgery.

## Conclusions

Young patients requiring a hip arthroplasty for treatment of a tumour or severe osteonecrosis of the femoral head are a very specific patient group of which little is known about the preferred treatment. Bipolar hemiarthroplasties are a reasonable option for this specific patient group with a survival rate, with conversion to THA or revision as endpoint, of 96% after 15 years, and 60% after 20 years. The advantage of 15 or more years before converting to a total hip, in our view, outweights the benefit of a total hip arthroplasty, especially because the acetabular component is at high risk of revision in this specific patient group. Furthermore, conversion to a THA is not difficult, with the hardened subchondral bone of the acetabulum as a possible positive factor influencing longer acetabular component survival after conversion. Because of the, in our experience, high conversion rate after unipolar hemiarthroplasties; we would not recommend this type of prosthesis in this patient group.

## Competing interest

No benefits in any form have been received or will be received from a commercial party related directly or indirectly to the subject of this article. The authors declare that they have no competing interests.

## Authors’ contributions

PWE acquisition of data, data analysis, writing. HJLH supervision, critical discussions, study design, reviewing. AHMT critical discussions, reviewing. All authors read and approved the final manuscript.

## Authors’ information

Pim W. van Egmond resident orthopaedic surgery.

Antonie H.M. Taminiau professor in orthopaedic oncology.

Huub J.L. van der Heide consultant orthopaedic surgeon.

## Pre-publication history

The pre-publication history for this paper can be accessed here:

http://www.biomedcentral.com/1471-2474/14/31/prepub
